# Decreased Virulence of Ross River Virus Harboring a Mutation in the First Cleavage Site of Nonstructural Polyprotein Is Caused by a Novel Mechanism Leading to Increased Production of Interferon-Inducing RNAs

**DOI:** 10.1128/mBio.00044-18

**Published:** 2018-08-21

**Authors:** Xiang Liu, Margit Mutso, Age Utt, Anni Lepland, Lara J. Herrero, Adam Taylor, Jayaram Bettadapura, Penny A. Rudd, Andres Merits, Suresh Mahalingam

**Affiliations:** a Institute for Glycomics, Griffith University, Gold Coast Campus, Southport, Queensland, Australia; b Institute of Technology, University of Tartu, Tartu, Estonia; University of Pittsburgh School of Medicine

**Keywords:** Ross river virus, Sindbis virus, alphavirus, interferons

## Abstract

Infection with Ross River virus (RRV) causes debilitating polyarthritis and arthralgia in individuals. Alphaviruses are highly sensitive to type I interferon (IFN). Mutations at the conserved P3 position of the cleavage site between nonstructural protein 1 (nsP1) and nsP2 (1/2 site) modulate type I IFN induction for both RRV and Sindbis virus (SINV). We constructed and characterized RRV-T48_A534V_, a mutant harboring an A534V substitution in the P1 position of the 1/2 site, and compared it to parental RRV-T48 and to RRV-T48_A532V_, SINV_I538_ and SINV_T538_ harboring different substitutions in the same region. A534V substitution resulted in impaired processing of RRV nonstructural polyprotein and in elevated production of replicase-generated pathogen-associated molecular pattern (PAMP) RNAs that induce expression of type I IFN. Both A532V and A534V substitutions affected synthesis of viral RNAs, though the effects of these closely located mutations were drastically different affecting mostly either the viral negative-strand RNA or genomic and subgenomic RNA levels, respectively. Synthesis of PAMP RNAs was also observed for SINV replicase, and it was increased by I538T substitution. In comparison to RRV-T48, RRV-T48_A534V_ was attenuated *in vitro* and *in vivo*. Interestingly, when type I IFN-deficient cells and type I IFN receptor-deficient mice were infected with RRV-T48 or RRV-T48_A534V_, differences between these viruses were no longer apparent. Compared to RRV-T48, RRV-T48_A534V_ infection was associated with increased upregulation of type I IFN signaling proteins. We demonstrate novel mechanisms by which the A534V mutation affect viral nonstructural polyprotein processing that can impact PAMP RNA production, type I IFN induction/sensitivity, and disease.

## IMPORTANCE

This study gives further insight into mechanisms of type I IFN modulation by the medically important alphaviruses Ross River virus (RRV) and Sindbis virus (SINV). By characterizing attenuated RRV mutants, the crucial role of amino acid residues in P1 and P3 positions (the first and third amino acid residues preceding the scissile bond) of the cleavage site between nsP1 and nsP2 regions was highlighted. The study uncovers a unique relationship between alphavirus nonstructural polyprotein processing, RNA replication, production of different types of pathogen-associated molecular pattern (PAMP) RNAs, type I IFN induction, and disease pathogenesis. This study also highlights the importance of the host innate immune response in RRV infections. The viral determinants of type I IFN modulation provide potential drug targets for clinical treatment of alphaviral disease and offer new approaches for rational attenuation of alphaviruses for construction of vaccine candidates.

## INTRODUCTION

Ross River virus (RRV) is a member of the *Togaviridae* family and the genus *Alphavirus*. It is the most widespread arbovirus in Australia and can be found throughout the South Pacific region. Each year in Australia, approximately 5,000 cases of RRV disease are reported (1). Several sporadic epidemics of RRV have occurred in the past few decades, the largest of which occurred from 1979 to 1980 and involved several countries, including Papua New Guinea, Fiji, Samoa, New Caledonia, and the Cook Islands (2, 3). Patients infected with RRV can develop polyarthritis, myalgia, and rash (4), and a subset of patients goes on to experience chronic arthralgia and debilitating pains that can last for months (5). There are currently no specific treatments or vaccines available for RRV.

The interferon (IFN) system is the first line of defense against invading microorganisms. The mechanisms by which host cells detect viruses, leading to triggering of the type I IFN system, differ according to cell type (6). Multiple regulatory proteins are involved in the induction of type I IFN and contribute to its antiviral effects, including Toll-like receptors (TLRs), interferon regulatory factor 7 (IRF7), 2′-5′-oligoadenylate synthetase, and the retinoic acid-inducible gene I product (RIG-I)-like helicase (RLH) family members. For Chikungunya virus (CHIKV) infection, both RLH and TLR family members are involved in the type I IFN response (7, 8). For Semliki Forest virus (SFV) and Sindbis virus (SINV) infection, RLH family members RIG-I and MDA5 play roles in type I IFN induction (9, 10). For RRV infection, Toll-like receptor 7 (TLR7) and MyD88 are reported to be responsible for antiviral protection (11).

Arboviruses have evolved different mechanisms to antagonize the type I IFN system (12–14). For Old World alphaviruses, such as SFV and SINV, this antagonism occurs via the activities of nonstructural proteins (nsPs) (13, 14). Host protein transcriptional/translational shutoff is one way of accomplishing this, as mutant viruses defective for shutoff induce more type I IFN (15). Furthermore, reports have shown that a mutation in the region corresponding to the junction of nonstructural protein 1 (nsP1) and nsP2 regions (referred to hereafter as 1/2 site) in nonstructural polyproteins P123/P1234 of SINV and RRV has a substantial effect on type I IFN induction independent of virus-mediated host shutoff (16). Mutations in nsP2 of SFV also affect type I IFN induction independently of host shutoff (13). This region has been identified as an important virulence factor for SFV (17) and SINV (18–22); however, the molecular mechanism(s) behind these effects has not been revealed.

The cleavage motif between nsP1 and nsP2 of RRV is R^P4^A^P3^G^P2^A^P1^↓G^P1′^V^P2′^V^P3′^E^P4′^ (Table 1), with cleavage occurring after the P1 Ala residue (23). The mutant virus designated RRV-T48_A532V_ has a substitution at the P3 position of the 1/2 site. This mutation resulted in higher levels of type I IFN induction in both L929 cells and type I IFN receptor knockout (IFNAR^−/−^) primary mouse embryonic fibroblasts (MEFs) (16). Moreover, increased levels of phosphorylated IRF3 were detected in RRV-T48_A532V_-infected L929 cells and were thought to be responsible for the enhanced expression of type I IFN. The data suggest that for RRV, the A532V mutation has an effect on the inductive phase of the type I IFN response (16).

**TABLE 1:** P4-to-P4′ residues of the 1/2 cleavage site of different alphaviruses and their strains

Virus (strain)	Amino acid position	Residue at the following position of the cleavage motif:							
		P4	P3	P2	P1	P1′	P2′	P3′	P4′
RRV-T48	531	R	A	G	A	G	V	V	E
SFV4	534	H	A	G	A	G	V	V	E
CHIKV	532	R	A	G	A	G	I	I	E
SINV_T538_ (S.A.AR86)	537	D	T	G	A	A	L	V	E
SINV_I538_ (TOTO1101)	537	D	I	G	A	A	L	V	E

In this study, we aimed to further characterize the molecular defect caused by mutations at the 1/2 site of RRV as well as mechanism(s) behind attenuation of RRV harboring such mutations. We took advantage of the fact that the Ala534 residue in the P1 position of the 1/2 site is highly conserved among the alphaviruses (Table 1). We demonstrate novel mechanisms by which the A534V mutation affects nonstructural polyprotein processing, resulting in an altered production of RNA PAMPs, including those derived from cellular RNAs, leading to enhanced IFN production. Furthermore, our findings link nonstructural polyprotein processing to viral pathogenesis.

## RESULTS

### RRV-T48_A534V_ is highly infectious and genetically stable.

The rescue efficiency of RRV-T48_A534V_, a mutant in which the conserved P1 Ala residue in the 1/2 site was substituted to Val, was compared to that of parental RRV-T48. Infectious-center assays (ICA) performed using BHK-21 cells revealed that both viruses were efficiently rescued (efficiencies of 9 × 10^4^ PFU/µg for RRV-T48_A534V_ and 5.59 × 10^4^ for PFU/µg for RRV-T48; no statistically significant difference was observed). Quantitative reverse transcription-PCR (qRT-PCR) analysis revealed that RRV-T48_A534V_ stock had a genome copy number/PFU ratio similar to that of RRV-T48, indicating that RRV-T48_A534V_ stock did not contain a significant excess of defective or noninfectious particles (see Fig. S1 in the supplemental material).

10.1128/mBio.00044-18.2 FIG S1  Viral genome copy number/PFU ratio in the obtained viral stock. Viral genomic copy number was determined from viral stock by qRT-PCR. The ratio of viral genome copy number to titer (PFU/ml) was determined and compared to wild-type virus (taken as 1). Obtained values are shown as the mean ± SEM for three independent experiments. No observed difference reached statistical significance (unpaired Mann-Whitney test). Download FIG S1, TIF file, 0.1 MB. Copyright © 2018 Liu et al. 2018 Liu et al. This content is distributed under the terms of the Creative Commons Attribution 4.0 International license.

**FIG 1:**
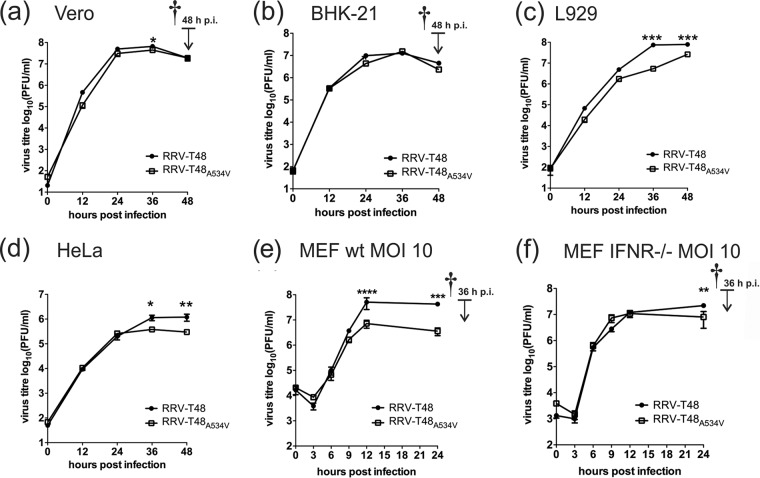
Multi- and single-step viral growth curves. (a to d) Growth kinetics of RRV-T48 or RRV-T48_A534V_ were analyzed in Vero (a), BHK-21 (b), L929 (c), and HeLa (d) cells. Cells were infected at an MOI of 0.1, and supernatants were collected at 12, 24, 36, and 48 h p.i. (e and f) Wild-type (wt) (e) and IFNAR^−/−^ (f) MEF cells were infected at an MOI of 10, and supernatants were collected at 3, 6, 9, 12, and 24 h p.i. Virus titers were analyzed by plaque assay. The time of death of the infected-cell culture is indicated by the dagger. Titers (in PFU/milliliter) are expressed as the means ± standard errors of the means (SEM) (error bars) from three independent experiments. Values that are significantly different by two-way ANOVA with Bonferroni *post hoc* test are indicated by asterisks as follows: *, *P* < 0.05; **, *P* < 0.01; ***, *P* < 0.001.

In Vero and BHK-21 cells, RRV-T48_A534V_ replicated to levels similar to those of RRV-T48 (Fig. 1a and b). To analyze the genetic stability of the A534V mutation, sequencing of amplified fragments corresponding to the region of the nsP1-nsP2 junction (nucleotides 1640 to 2500 of the RRV-T48 genome) was performed. It revealed the presence of the A534V mutation in all clones analyzed; no secondary (adaptive or pseudoreversion) mutations were found.

### A534V mutation inhibits *in vitro* replication of RRV in type I IFN-positive cells.

To determine the effect of the A534V mutation on RRV replication *in vitro*, multistep growth curves at a multiplicity of infection (MOI) of 0.1 on BHK-21, Vero, HeLa, and L929 cells were obtained. In BHK-21 and Vero cells, only small differences between the two viruses were observed at some time points (Fig. 1a and b). In contrast, RRV-T48_A534V_ grew to significantly lower final titers than RRV-T48 did in type I IFN-competent L929 and HeLa cells (Fig. 1c and d).

To verify the effect of an intact type I IFN system, a growth curve experiment was performed using wild-type (wt) and IFNAR^−/−^ MEFs. At an MOI of 0.1, both viruses replicated faster in IFNAR^−/−^ MEFs than in wt MEFs; the difference was most prominent at earlier (12 and 24 h postinfection [p.i.]) time points (Fig. S2a and b). No difference between growth of RRV-T48 and RRV-T48_A534V_ was observed in IFNAR^−/−^ MEFs (Fig. S2b). In wt MEFs, growth of RRV-T48_A534V_ was slower than that of RRV-T48; however, the differences did not reach statistical significance with both viruses replicating to similar final titers. All cells in the infected cultures were dead by 48 h p.i. (Fig. S2a and b). Similar data were obtained using a lower MOI (0.01) (Fig. S2c and d). This confirms that the A534V mutation does not block the ability of RRV to inhibit type I IFN-mediated signaling from infected to noninfected bystander cells, as the latter acquired no protection against subsequent infection by virions released from initially infected cells.

10.1128/mBio.00044-18.3 FIG S2  Multistep growth curves in MEF cells. Growth kinetics of RRV-T48 (black circles) or RRV-T48_A534V_ (empty squares) were analyzed in wt (a and c) and IFNAR^−/−^ (b and d) MEF cells. Cells were infected at an MOI of 0.1 (a and b) or MOI of 0.01 (c and d), and supernatants were collected at 12, 24, 36, and 48 h p.i. Virus titers were analyzed by plaque assay. The dagger indicates the time of death of the infected-cell culture. Titers are expressed as the mean PFU ± SEM for three independent experiments. No statistically significant difference between viruses was observed by two-way ANOVA with Bonferroni *post hoc* test. Download FIG S2, TIF file, 0.3 MB. Copyright © 2018 Liu et al. 2018 Liu et al. This content is distributed under the terms of the Creative Commons Attribution 4.0 International license.

As growth of both viruses was faster in IFNAR^−/−^ MEFs, it was hypothesized that RRV replication in wt MEFs is likely slowed down by a type I IFN-mediated autocrine signal amplification loop. To test this, single-step growth curves (at an MOI of 10) were obtained using wt and IFNAR^−/−^ MEF cells. At a high MOI, RRV-T48 displayed a clear growth advantage in wt MEFs and reached significantly higher titers at 12 and 24 h p.i. (Fig. 1e). In contrast, growth curves for both viruses in IFNAR^−/−^ MEFs remained similar (Fig. 1f). Thus, RRV-T48_A534V_ was significantly attenuated in cells with an intact type I IFN system and that this attenuation was likely caused by the antiviral effects of type I IFN on the infected cells themselves.

### RRV-T48_A534V_ has higher sensitivity to IFN-β and induces higher levels of type I IFNs.

**FIG 2:**
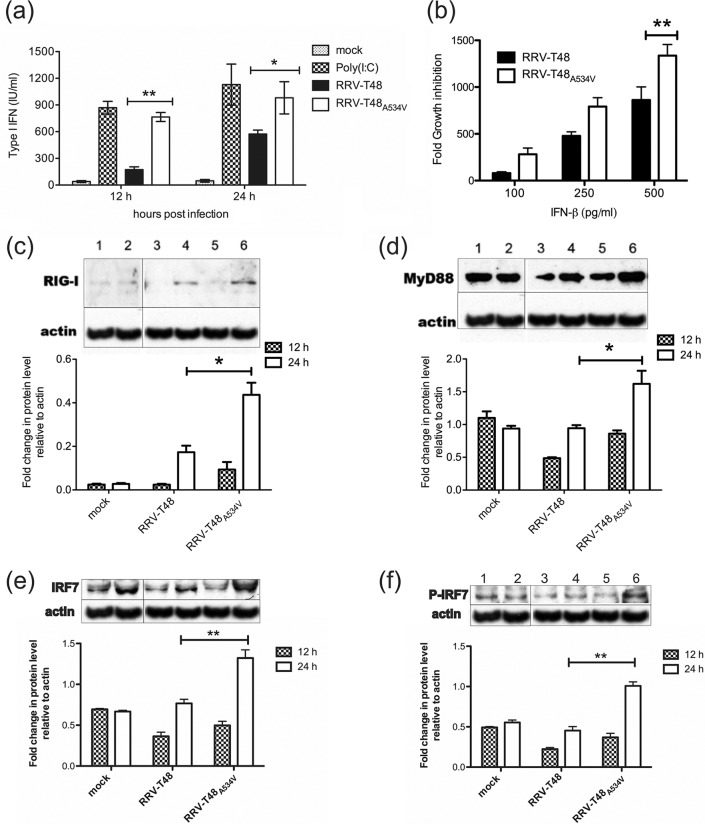
Type I IFN induction and sensitivity of RRV-T48 and RRV-T48_A534V_ and comparison of expression levels of type I IFN signaling proteins in RRV-T48- and RRV-T48_A534V_-infected L929 cells. (a) L929 cells were infected with RRV-T48 or RRV-T48_A534V_ at an MOI of 0.1. Transfection with poly(I · C) was used as a positive control for type I IFN induction. Supernatants were collected at 12 h and 24 h p.i./p.t. (posttransfection), and type I IFN levels were determined by IFN bioassay. (b) Vero cells were infected by either RRV-T48 or RRV-T48_A534V_ at an MOI of 0.1. Infected cells were treated with increasing concentrations of human IFN-β. The cell culture supernatants were collected at 24 h p.i. and analyzed using plaque assay. The sensitivity of RRV-T48 and RRV-T48_A534V_ to type I IFN is represented by a decrease in viral titer resulting from IFN-β treatment. (c to f) For analysis of expression levels of type I IFN signaling proteins, L929 cells were mock infected or infected with RRV-T48 or RRV-T48_A534V_ at an MOI of 0.1. Cell lysates were collected at 12 h and 24 h p.i. and analyzed by Western blotting using antibodies recognizing RIG-I (c), MyD88 (d), IRF7 (e), and phospho-IRF7 (P-IRF7) (f). β-Actin was used as loading control. Bands were quantified using ImageJ software. Results are represented as fold change over β-actin. In all panels, error bars represent SEM from three independent experiments (*, *P* < 0.05; **, *P* < 0.01 using two-way ANOVA with Bonferroni *post hoc* test).

In order to compare the levels of type I IFN secreted in response to infection by RRV-T48 and RRV-T48_A534V_, L929 cells were infected at an MOI of 0.1. RRV-T48_A534V_ induced significantly more type I IFN than RRV-T48 did (Fig. 2a). To analyze the type I IFN sensitivity of RRV-T48_A534V_, Vero cells (which are unable to produce type I IFN but can react to exogenous IFN) were infected with RRV-T48 or RRV-T48_A534V_ and then treated with increasing amounts of human beta interferon (IFN-β). Replication of both RRV-T48 and RRV-T48_A534V_ was strongly reduced by IFN-β treatment. However, the growth inhibition of RRV-T48_A534V_ was always more prominent than that of RRV-T48**,** and this difference was statistically significant at 500 pg/ml IFN-β (Fig. 2b). Taken together, these results show that, compared to RRV-T48, RRV-T48_A534V_ induced higher levels of type I IFN and had an increased sensitivity to antiviral effects of IFN-β. The differences between the two viruses were, however, rather small (no more than severalfold [Fig. 2b]), which is consistent with the relatively slight attenuation of mutant virus growth in type I IFN-competent cells (Fig. 1c to e).

### RRV-T48_A534V_ infection results in upregulation of RIG-I, MyD88, and IRF7 expression.

To identify genes involved in antiviral signaling, L929 cells were infected with RRV-T48 or RRV-T48_A534V_ at an MOI of 0.1 and were analyzed by Western blotting for expression of the major signaling proteins in the type I IFN induction pathways. At 24 h p.i., the levels of RIG-I (Fig. 2c) and MyD88 (Fig. 2d) were significantly upregulated by infection with RRV-T48_A534V_ compared to infection with RRV-T48. Similarly, IRF7 expression and its phosphorylation were also significantly higher in RRV-T48_A534V_-infected cells (Fig. 2e and f). In contrast, no significant differences in the expression of TRAF3, TLR7, iKK-*i*, TBK1, MDA5, or IPS-1 were observed between RRV-T48- or RRV-T48_A534V_-infected cells (Fig. S3).

10.1128/mBio.00044-18.4 FIG S3  Comparison of expression levels of type I IFN signaling proteins in RRV-T48- and RRV-T48_A534V_-infected L929 cells. For expression levels of type I IFN signaling proteins, cells were mock infected or infected with RRV-T48 or RRV-T48_A534V_ at an MOI of 0.1. Cell lysates were collected at 12 h and 24 h p.i. and analyzed by Western blotting using antibodies recognizing TRAF3 (a), TLR7 (b), IKK-*i* (c), TBK1 (d), MDA5 (e), and IPS-1 (f). β-Actin was used as a loading control. Bands were quantified using ImageJ software; results are represented as fold change over β-actin. Error bars represent SEM from three independent experiments. No statistically significant difference between viruses was observed by two-way ANOVA with Bonferroni *post hoc* test. Download FIG S3, TIF file, 1.4 MB. Copyright © 2018 Liu et al. 2018 Liu et al. This content is distributed under the terms of the Creative Commons Attribution 4.0 International license.

### The A534V mutation slows down processing of RRV nonstructural polyprotein and delays virus-induced shutoff of host translation.

**FIG 3:**
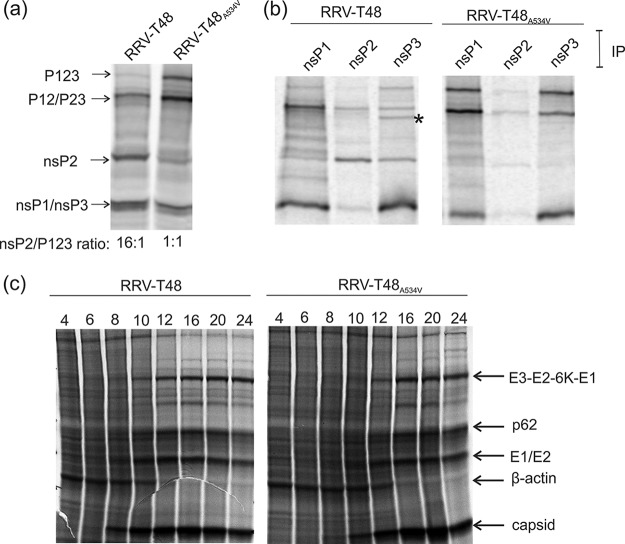
Processing of RRV-T48 and RRV-T48_A534V_ nonstructural polyproteins and analysis of virus-induced translational shutoff. (a and b) RRV-T48 and RRV-T48_A534V_ nonstructural polyproteins and their processing products were obtained using the TNT SP6 rabbit reticulocyte system and plasmids containing the RRV-T48- or RRV-T48_A534V_-infected cell DNAs. (a) The *in vitro*-translated nonstructural polyproteins and the products of their processing were denatured by boiling in 1% SDS. (b) Products of *in vitro* translation were immunoprecipitated (IP) using antibodies against nsP1, nsP2, and nsP3 of SFV. The asterisk indicates the position of the P34 polyprotein. (c) BHK-21 cells were infected at an MOI of 10 with RRV-T48 or RRV-T48_A534V_. At 4, 6, 8, 10, 12, 16, 20, and 24 h p.i., cells were labeled with medium containing 50 µCi [^35^S]Met and [^35^S]Cys. After labeling, cells were lysed in SDS loading buffer. In panels a to c, labeled proteins were separated by SDS-PAGE and visualized/quantified with a Typhoon imager. Data from one of two reproducible independent experiments are shown in each of the panels.

**FIG 4:**
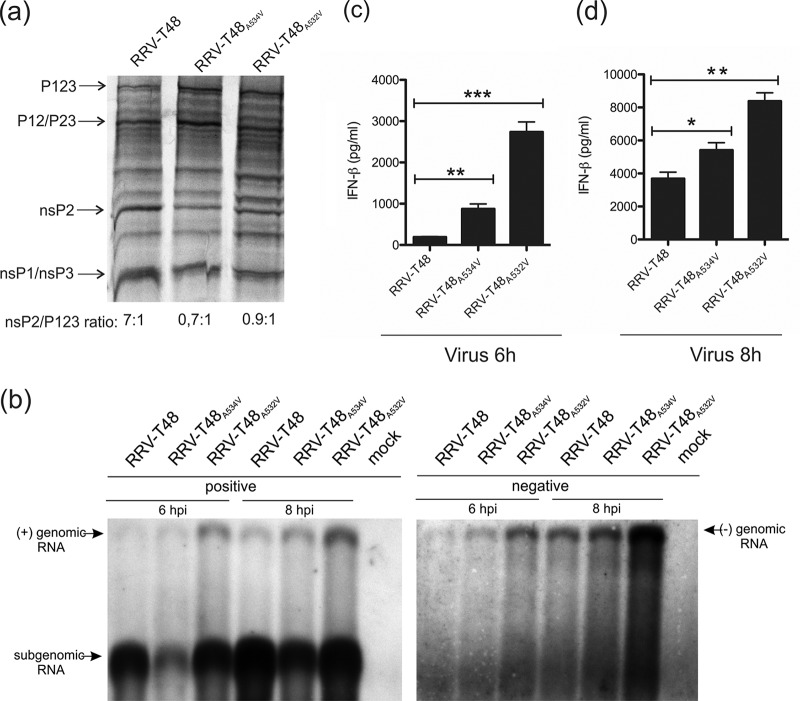
Effects of mutations in the P3 and P1 positions of the 1/2 site on RRV nonstructural polyprotein processing and RNA replication. (a) Processing of RRV-T48, RRV-T48_A532V_ and RRV-T48_A534V_ nonstructural polyproteins was analyzed as described in the legend to Fig. 3a. (b to d) BHK-21 cells were infected with RRV-T48, RRV-T48_A532V_ or RRV-T48_A534V_ at an MOI of 10. At 6 or 8 h p.i., cells were lysed, and total RNA was isolated. (b) Northern blot analysis. RNAs isolated from infected cells were separated by electrophoresis in agarose gels, transferred to a Hybond-N+ filter, and detected with probes complementary to positive and negative strands of RRV RNAs. (c and d) One microgram of each RNA sample was used for transfection of Cop5 cells; at 24 h p.t., the amount of IFN-β in the cell supernatant was determined. IFN-β amounts are expressed as the means ± SEM from three experiments performed in parallel (*, *P* < 0.05; **, *P* < 0.01; ***, *P* < 0.001 using Student’s two-tailed unpaired *t* test). Data from one of two reproducible independent experiments are shown in each of the panels.

The A534V substitution may hamper the release of nsP2, known to be the major antagonist of type I IFN for Old World alphaviruses (13–15). The processing of RRV nonstructural polyproteins was analyzed using an *in vitro* transcription/translation system. Processing of RRV-T48_A534V_-encoded P123 (due to an in-frame stop codon, this is the major nonstructural polyprotein of RRV) was found to be less efficient than that of P123 encoded by RRV-T48; stability of P123 itself and its processing products P12 and/or P23 was increased, while the release of mature nsP2 was diminished (Fig. 3a). As a result, the ratio of mature nsP2 to P123 precursor was ≥10-fold lower for RRV-T48_A534V_ than for RRV-T48 (Fig. 3a and 4a).

To confirm the identities of polyprotein processing intermediates and mature nsPs, the products of *in vitro* translation were immunoprecipitated using polyclonal antisera against nsP1, nsP2, and nsP3 of the related SFV. Immunoprecipitation with anti-nsP1 confirmed the increased stability of P123 and P12 of RRV-T48_A534V_ and concomitant reduction of release of free nsP1 (Fig. 3b). Immunoprecipitation also confirmed a clear reduction in the amount of mature nsP2 for RRV-T48_A534V_ and that the largest detected polyprotein was P123; it also revealed an increased stability of P23 and concomitant reduction of the amount of released nsP3 for RRV-T48_A534V_. Interestingly, the A534V mutation resulted in a minor polyprotein, which is likely to be P34, becoming almost undetectable (Fig. 3b), suggesting accelerated release of nsP4. Taken together, the A534V mutation delays cleavage at the 1/2 and 2/3 sites but has no negative effect on processing at the 3/4 site.

RRV-T48_A532V_ has been reported to cause complete, but delayed, shutoff of host cell translation in BHK-21 cells (16). When a similar experiment was performed with RRV-T48 and RRV-T48_A534V_, it was found that RRV-T48_A534V_ was also capable of inducing complete (or nearly complete) shutoff of host cell translation. Only a minor delay (around 2 h compared to RRV-T48), which may be attributed to slower release of nsP2 from the polyprotein harboring the mutation in the 1/2 site (Fig. 3a), was observed (Fig. 3c).

### The A534V mutation suppresses synthesis of RRV subgenomic RNA and increases synthesis of type I IFN-inducing PAMP RNAs.

As the properties of RRV-T48_A534V_ were similar to those reported for RRV-T48_A532V_ (16), we hypothesized that the two mutant viruses share a similar molecular defect responsible for enhanced induction of the type I IFN response. We compared nonstructural polyprotein processing of RRV-T48 and RRV-T48_A534V_ with that of RRV-T48_A532V_. Delayed processing was observed for RRV-T48_A532V_ (Fig. 4a). In comparison with RRV-T48_A534V_, the defect was slightly but reproducibly less pronounced, suggesting that the Val residue in the P1 position of the 1/2 site has a greater impact on nonstructural polyprotein processing than the same residue in the P3 position.

Slowdown of P123 processing should result in increased amounts of alphavirus early replicase, which consists of P123 and nsP4 subunits and synthesizes viral negative-strand RNAs (24). Therefore, we analyzed viral RNA synthesis in BHK-21 cells infected at a high MOI (10). Compared to RRV-T48, RRV-T48_A532V_ had a prominent increase of both negative- and positive-strand genomic RNA synthesis, while the synthesis of subgenomic (SG) RNA was not detectably affected. In contrast, RRV-T48_A534V_ had a prominent defect in SG RNA synthesis, while the levels of genomic-length RNAs were only slightly higher than those for RRV-T48 (Fig. 4b).

Negative-strand RNAs of alphaviruses lack a 5′ cap structure and exist as double-stranded RNA (dsRNA) duplexes (called replicative forms) with positive-strand RNAs (25). These properties make them PAMP RNAs that are recognized by cellular pathogen recognition receptors (PRRs) (9). As the A532V mutation in RRV-T48 was suggested to affect the inductive phase of the type I IFN response (16), we hypothesized that hugely increased production of viral negative-sense strands (Fig. 4b) may represent the primary reason for elevated type I IFN production in RRV-T48_A532V_-infected cells. To verify this directly, we transfected type I IFN-competent murine fibroblast (Cop5) cells with total RNAs isolated from BHK-21 cells infected with RRV-T48, RRV-T48_A534V_, or RRV-T48_A532V_ to activate IFN production; all RNAs used in this experiments were UV inactivated prior to transfection. Consistent with our hypothesis, total RNA isolated from RRV-T48_A532V_-infected BHK-21 cells induced much higher levels of IFN-β production than RNA from RRV-T48-infected cells. The difference was especially prominent (>10-fold) for RNAs isolated at 6 h p.i. (Fig. 4c); by 8 h p.i., the difference was smaller (≈2-fold) but remained highly significant (Fig. 4d). This finding strongly suggests that excessive negative-strand RNA synthesis, caused by increased stability of the early RNA replicase, is indeed the main reason for elevated type I IFN induction by RRV-T48_A532V_. RNA from RRV-T48_A534V_-infected cells also induced significantly more IFN-β than RNA isolated from RRV-T48-infected cells. Although the difference between viruses was less pronounced (up to fivefold at 6 h p.i.; Fig. 4c), the enhanced ability of RNA from RRV-T48_A534V_-infected cells to induce type I IFN production is unlikely to be solely due to the small increase in the amount of viral negative-strand RNAs (Fig. 4b).

### The Thr residue in the P3 position of the 1/2 site of SINV also slows down the processing of P123.

Interestingly, for SINV, it has been reported that slow processing of P123 caused by the Thr residue (residue 538 of nsP1) in the P3 position of the 1/2 site (19) led to reduced induction of type I IFN both *in vitro* and *in vivo* (16), which is the reverse for RRV. To investigate these differences from RRV, we used SINV TOTO1101 (referred to hereafter as SINV_I538_ as it has an Ile538 residue) to generate SINV_T538_ and performed the same set of experiments as were done with RRV-T48 and its mutants.

**FIG 5:**
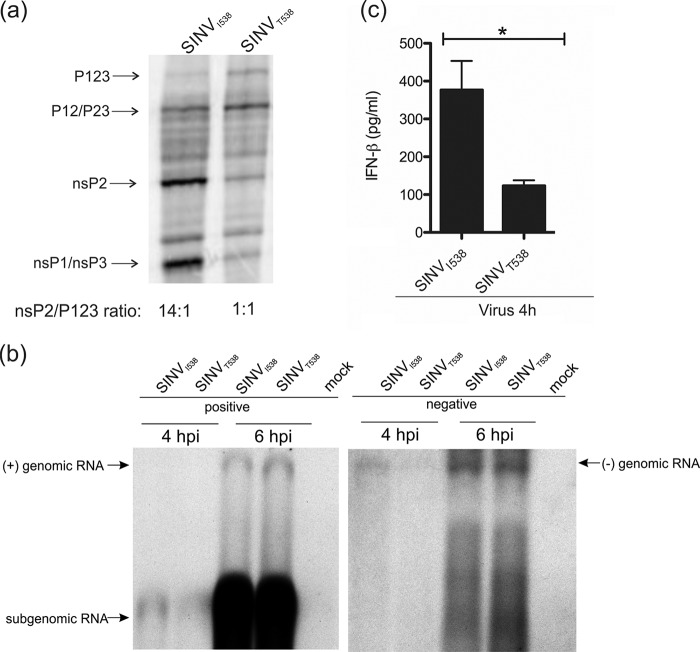
The Thr residue in the P3 position of the 1/2 site of SINV slows down P123 processing but does not increase production of PAMP RNAs in infected BHK-21 cells. (a) Processing of SINV_I538_ and SINV_T538_ nonstructural polyproteins was analyzed as described in the legend to Fig. 3a. (b and c) BHK-21 cells were infected with SINV_I538_ or SINV_T538_ at an MOI of 10. At 4 or 6 h p.i., cells were lysed, and total RNA was isolated. (b) Northern blot analysis was performed as described in the legend to Fig. 4b except that probes revealing positive and negative strands of SINV RNAs were used. (c) One microgram of each RNA sample was used for transfection of Cop5 cells. The amount of IFN-β in the cell supernatant at 24 h p.t. was determined. IFN-β amounts are expressed as the means ± SEM from three experiments performed in parallel (*, *P* < 0.05 using Student’s two-tailed unpaired *t* test). Data from one of two reproducible independent experiments are shown in each of the panels.

**FIG 6:**
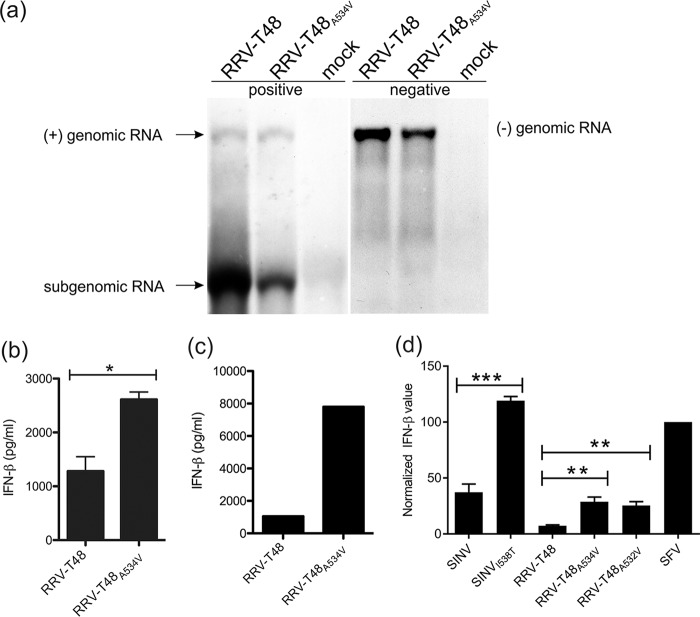
Replicases of RRV and SINV synthesize PAMP RNAs using cellular templates. BHK-21 cells were infected with RRV-T48 and RRV-T48_A534V_ at an MOI of 1. At 6 h p.i., cells were lysed and total RNA was isolated. (a) Northern blot analysis was performed as described in the legend to Fig. 4b. Data from one of two reproducible independent experiments are shown. (b) One microgram of each RNA sample was used for transfection of Cop5 cells. The amount of IFN-β in the cell supernatant at 24 h p.t. was determined. IFN-β levels are expressed as means plus SEM from three independent experiments (*, *P* < 0.05 using Student’s two-tailed unpaired *t* test). (c) Poly(A)^−^ fraction was obtained from isolated RNAs by removal of poly(A)^+^ RNAs, including viral dsRNA replication forms. One microgram of each RNA sample was used for transfection of Cop5 cells. The amount of IFN-β in the cell supernatant at 24 h p.t. was determined. Data from one of two reproducible independent experiments are shown. (d) Cop5 cells were transfected with plasmids designed to express wild-type and mutant forms of replicases of SFV, RRV, and SINV; the amount of IFN-β in supernatant was determined at 48 h p.t. Values obtained for cells transfected with RRV or SINV replicase expression plasmids were normalized to values obtained for cells transfected with plasmid expressing SFV4 replicase (taken as 100). Normalized values are shown as the means plus SEM from three independent experiments (**, *P* < 0.01; ***, *P* < 0.001 using two-way ANOVA with Bonferroni *post hoc* test).

We first confirmed that the Thr538 residue does cause delay in SINV P123 processing. The observed reduction of cleavage efficiency was similar to that caused by the A534V mutation in RRV (compare Fig 5a and 3a). Compared to RRV, SINV grew more rapidly and to much higher final titers, possibly due to cell culture adaptation of the TOTO1101 strain. The effect of the I538T substitution on synthesis of SINV RNAs was relatively mild. We did observe the earlier activation of SG RNA synthesis for SINV_I538_ (Fig. 5b). However, we also observed that the same virus produced more negative-strand RNA by 4 h p.i. (Fig. 5b), which is different from findings made using the S.A.AR86 strain of SINV where Ile538 did not increase genome-length RNA synthesis (19). Thus, the elevated negative-strand RNA synthesis may be a strain-specific phenomenon. Transfection of total RNA isolated at 4 h p.i. from SINV_I538_-infected cells into Cop5 reporter cells induced significantly more type I IFN than did the RNA isolated from SINV_T538_-infected cells (Fig. 5c). This may be due to the elevated levels of negative-strand RNAs (Fig. 5b) and is consistent with reports that SINV strains (or mutants) harboring the Thr538 residue produce less type I IFN than strains with the Ile538 residue (16, 20). There the results were opposite to the findings made for RRV and its mutants, where slow processing of P123 was always associated with significantly elevated production of type I IFN-inducing RNAs (Fig. 4c and d; Fig. 6b). No difference between type I IFN-inducing capability of RNAs isolated from SINV_I538_- and SINV_T538_-infected cells at later time points was observed (data not shown).

### RRV-T48_A534V_ and its replicase produce an excess of type I IFN-inducing PAMP RNAs made using cellular templates.

To further investigate the reason(s) for the enhanced ability of RNA from RRV-T48_A534V_-infected cells to induce type I IFN production, we performed infection of BHK-21 cells with RRV-T48 and RRV-T48_A534V_ at an MOI of 1. This MOI was chosen, since at a lower MOI, the parental virus has a slight growth advantage over the mutant virus (Fig. 1b). Northern blotting confirmed that under these conditions, RRV-T48 synthesized more positive- and negative-strand RNAs than RRV-T48_A534V_ did (Fig. 6a).

Aliquots of the RNA samples shown in Fig. 6a were used to transfect Cop5 cells. Consistent with previous data, RNA from RRV-T48_A534V_-infected cells induced higher IFN-β levels than RNA from RRV-T48-infected cells (Fig. 6b). Here, however, the elevated IFN-β induction could not be attributed to an increased amount of negative-strand RNAs/replicative forms referred to hereafter as “viral PAMP RNAs” (Fig. 6a). Thus, our data demonstrate the presence of other type(s) of virus-generated PAMP RNAs in RRV-T48_A534V_-infected cells.

Alphaviruses can synthesize noncapped positive-strand RNAs (26) that may be recognized by RIG-I (9). Though we cannot exclude the possibility that such RNAs affect type I IFN induction by RRV-T48_A534V_, it was considered unlikely, as this mutant virus synthesized positive-strand RNAs (especially SG RNAs) in reduced amounts (Fig. 6a). It was also recently demonstrated that the majority of PAMP RNAs in SFV-infected cells are short nonpolyadenylated dsRNAs with 5′ triphosphate that are synthesized by SFV replicase activity on cellular RNA templates (10). Therefore, we hypothesized that such PAMP RNAs (hereafter referred as “replicase PAMP RNAs”) may also be present in RRV-infected cells and that it was their production that was activated by the A534V mutation.

In contrast to replicase PAMP RNAs, all viral PAMP RNAs have poly(A) tails and thus can be removed from total RNA samples using oligo(dT) resin (10). By using this method, we obtained poly(A)^−^ fractions from previously analyzed total RNA samples (Fig. 6a and b) and used these to transfect Cop5 cells. Poly(A)^−^ RNA isolated from RRV-T48_A534V_-infected BHK-21 cells consistently induced much higher levels of IFN-β than similar RNA obtained from RRV-T48-infected cells (Fig. 6c). These data are consistent with synthesis of replicase PAMP RNAs in RRV-infected BHK-21 cells and confirm the hypothesis that synthesis of such RNAs was indeed enhanced by the A534V mutation.

Finally, the ability of RRV replicase to synthesize replicase PAMP RNAs in the absence of replication-competent (viral) template RNA was confirmed using a plasmid-based expression system. Consistent with a previous report (10), Cop5 cells transfected with plasmid expressing replicase from SFV4 produced a high level of IFN-β; this allowed us to use this plasmid for normalization of levels of IFN-β measured for cells transfected with plasmids expressing replicases of RRV-T48, RRV-T48_A534V_, RRV-T48_A532V_, SINV_I538_, and SINV_T538_. Compared to SFV4 replicase, expression of RRV-T48 replicase induced much lower (but still clearly detectable) levels of IFN-β (Fig. 6d). Both A534V and A532V mutations increased this ability significantly (Fig. 6d). Interestingly, SINV_I538_ replicase also induced expression of IFN-β and did it more efficiently than RRV-T48 replicase. The presence of the Thr538 residue further increased IFN-β production by SINV replicase (Fig. 6d). However, in infected cells, SINV_T538_ clearly synthesized less PAMP RNA than SINV_I538_ did (Fig. 5c), suggesting that for cell culture-adapted SINV, which has very rapid RNA replication, the impact of elevated levels of replicase PAMPs was small compared to that of viral PAMPs synthesized in excess by SINV_I538_ (Fig. 5b).

### RRV-T48_A534V_ produces lethal disease in IFNAR^−/−^ mice.

**FIG 7:**
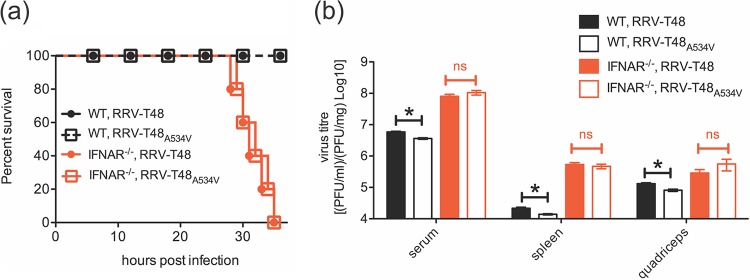
RRV-T48_A534V_ infection is not attenuated in IFNAR^−/−^ mice. (a) Groups of 20-day-old C57BL/6 wild-type (WT) and IFNAR^−/−^ mice (five animals in each group) were infected s.c. with 10^4^ PFU of RRV-T48 or RRV-T48_A534V_. Animals were monitored every 6 h during the first 24 h p.i. and every 2 h thereafter. (b) Groups of 20-day-old WT and IFNAR^−/−^ C57BL/6 mice (five animals in each group) were infected s.c. with 10^4^ PFU of RRV-T48 or RRV-T48_A534V_. Mice were sacrificed at 24 h p.i., and sera, spleens, and quadriceps were collected. Viral titers were assessed by plaque assay. The limit of detection for plaque assay is 30 PFU/ml for serum samples, 0.6 PFU/mg for spleen samples, and 1 PFU/mg for quadriceps samples. Statistical significance of the different values observed for RRV-T48- and RRV-T48_A534V_-infected mice were analyzed and presented as follows: *, *P* < 0.05 using Student’s two-tailed unpaired *t* test; ns, not significant. All experiments were repeated twice with reproducible results.

Mice infected with RRV-T48_A534V_ develop less severe disease and display reduced myositis compared to mice infected with parental RRV-T48 (Fig. S4). In order to analyze whether *in vivo* attenuation of RRV-T48_A534V_ is due to increased type I IFN production and/or sensitivity, groups of wild-type (WT) and IFNAR^−/−^ C57BL/6 mice were infected subcutaneously (s.c.) with 10^4^ PFU of RRV-T48 or RRV-T48_A534V_ and monitored for disease signs. IFNAR^−/−^ mice infected with either virus reached the experimental humane endpoint between 28 and 36 h p.i., in contrast to WT mice, which all survived infection (Fig. 7a). In WT mice, the titers of RRV-T48_A534_ in sera, spleens, or quadriceps were significantly lower than those for RRV-T48. In IFNAR^−/−^ mice, titers of both viruses were always higher than in WT C57BL/6 mice, and importantly, no statistically significant differences between the titers of RRV-T48_A534V_ compared to those of RRV-T48 were observed (Fig. 7b). These data confirm that type I IFN is crucial in controlling replication of both RRV-T48 and RRV-T48_A534V_ *in vivo* and that the impact of the type I IFN system is greater on virus harboring the A534V mutation.

10.1128/mBio.00044-18.5 FIG S4  RRV-T48_A534V_ infection causes mild disease in C57BL/6 mice. Groups of 20-day-old wt C57BL/6 mice (five animals per group) were injected s.c. with 10^4^ PFU RRV-T48 or RRV-T48_A534V_. Control mice were injected with PBS. (a and b) Mouse weights (a) and clinical scores (b) were monitored daily. Mice were scored according to hind limb strength and onset of hind limb dysfunction. (c) Quadriceps samples were collected on days 6, 10, and 15 p.i., fixed in 4% paraformaldehyde, embedded in paraffin, cut into 5-µm-thick sections, and stained with hematoxylin and eosin (H&E). Images are shown at a magnification of ×100. For statistical analysis, cell infiltrates were quantified using ImageJ software; results are shown in panel d. In panels a, b, and d, all values represent the mean ± SEM with five mice per group. Statistical significance of differences observed between RRV-T48- and RRV-T48_A534V_-infected mice were analyzed and presented as follows: **, *P* < 0.01 using two-way ANOVA with Bonferroni posttest (a); *, *P* < 0.05 using nonparametric Mann-Whitney test (b); *, *P* < 0.05, **, *P* < 0.01, ***, *P* < 0.001 using two-way ANOVA with Bonferroni posttest (d). All experiments were repeated twice with reproducible results. Download FIG S4, TIF file, 1.7 MB. Copyright © 2018 Liu et al. 2018 Liu et al. This content is distributed under the terms of the Creative Commons Attribution 4.0 International license.

## DISCUSSION

The interaction of RRV with the host type I IFN system is not well understood. For other alphaviruses, links between nonstructural polyprotein processing and virus *in vitro/in vivo* phenotype have emerged. SINV mutants lacking P123 processing were unable to downregulate host type I IFN responses (27). A similar effect has been described for SFV with delayed processing at the 1/2 site (28). Furthermore, neurovirulence of SFV and SINV in mouse models is associated with slower 1/2 site processing and higher stability of P123 due to suboptimal P4 (SFV) or P3 (SINV) residues in the 1/2 site (17, 19). However, the precise mechanisms by which processing affects so many different properties of alphaviruses are not well understood.

In this study, we demonstrated that for RRV, the P1 Ala-to-Val substitution slows down 1/2 site processing and results in stabilization of P123 and P12 (Fig. 3a and b); the same substitution at the P3 position resulted in a very similar effect (Fig. 4a). Mutation in the 1/2 site affected processing at the other two. Stabilization of P23 is most likely a consequence of decreased levels of nsP2, as the cleavage of alphavirus P23 occurs in *trans* and requires prior release of the N terminus of nsP2 (29, 30). Reduced amounts of P34 for RRV-T48_A534V_ may indicate that increased stability of the P123 region of the P1234 polyprotein (Fig. 3a and b) facilitates *in cis* cleavage of the 3/4 site, and as a result, less P34 is formed. Thus, a mutation in the P1 position of the 1/2 site has a profound effect on overall processing of RRV nonstructural polyprotein and therefore should have an impact on biological properties of mutant virus.

Alphaviruses such as Venezuelan equine encephalitis virus (VEEV), CHIKV, and SINV are able to antagonize type I IFN by disrupting STAT signaling (20, 31, 32). In addition, SINV also targets IPS-1 to interfere with type I IFN induction pathways (22). The A532V mutation in RRV-T48_A532V_ results in increased IRF3 phosphorylation, which indicates that upstream effectors of the type I IFN induction pathway may also play a role in type I IFN induction and sensitivity (16). The finding that A534V and A532V mutations result in a slowdown of the release of nsP2, the major suppressor of antiviral responses for Old World alphaviruses (13–15, 31, 32), is consistent with this scheme. Free nsP2 in the nucleus counteracts the IFN response (13, 14) by blocking the Jak-STAT signaling pathway (31, 32) and/or causing transcriptional shutoff of IFN-stimulated genes (15). However, the ability of RRV-T48_A534V_ to cause complete translational shutoff of infected cells and its efficient spread in cell cultures capable of type I IFN signaling led us to consider that delayed release of nsP2 is unlikely to be the primary mechanism behind enhanced type I IFN stimulation of RRV-T48_A534V_.

The most basic process in alphavirus infection is RNA synthesis; among other things, it is responsible for production of PAMP RNAs recognized by host PRRs. As P123 of RRV-T48_A534V_ and RRV-T48_A532V_ have increased stability (Fig. 3a and 4a), it was logical to assume that their P123-nsP4 (early replicase) complexes should be more active than those of parental RRV-T48. Our data clearly demonstrate that synthesis of different viral RNAs was indeed prominently affected by mutations in the 1/2 site. Surprisingly, however, two very similar mutants (RRV-T48_A534V_ and RRV-T48_A532V_) had very different levels of negative-strand RNA synthesis, with the enhancing effect of the A532V mutation being clearly more prominent (Fig. 4b). Thus, subtle differences in P123 processing (and/or different location of the mutation-introduced Val residue) resulted in different phenotypes.

Alphavirus RNA replication forms are long dsRNAs and represent the best known PAMPs produced during the course of virus infection. However, the levels of these PAMP RNAs could not explain strongly elevated type I IFN production during RRV-T48_A534V_ infection. This conundrum was solved by the observation that RRV-T48 produces an additional type(s) of RNA PAMPs. Although we did not characterize in detail the novel PAMP RNAs made by RRV, they share the two most important characteristics of SFV replicase PAMP RNAs described earlier (10): they are nonpolyadenylated and can be made by viral replicase in the absence of replication-competent RNA template. PAMP RNAs of this type are also made by replicases of distantly related hepatitis C virus (10) and picornaviruses (33).

dsRNA serves as a common nominator for both viral and replicase PAMP RNAs. The only form of alphavirus replicase complex clearly shown to synthesize dsRNAs is an early replicase, which is the precise complex we found to be stabilized by A534V and A532V mutations. Consistent with this, these mutant viruses indeed produced more PAMP RNAs in the viral infection and replicase context; the same was observed for replicases of SINV_I538_ and SINV_T538_. The levels of IFN-β induced by the production of replicase PAMPs were different for different viruses and their mutants. RRV replicases were clearly less potent inducers than replicases of SFV and SINV_T538_. Nevertheless, there was a clear trend that replicases of viruses with slower processing of P123 induced significantly higher IFN-β levels than replicases of their counterparts having faster processing (Fig. 6d). Although the levels of IFN-β in our assay system may be somewhat modulated by the timing of nsP2 (which is expressed as part of replicase) release, it is likely that the amount of IFN-β directly correlates with the amount of PAMP RNAs in transfected cells as was observed for RNAs isolated from virus-infected cells (Fig. 6a and c). This also implies that production of replicase PAMPs is a property of alphavirus short-lived early (negative-strand) replicase, rather than stable late (positive-strand) replicase.

A previous study comparing variants of SINV and RRV harboring substitutions in the P3 position of the 1/2 site concluded that for both viruses attenuated variants induced excessive amounts of type I IFN (16). It should be noted the term “attenuated” in that study actually applied only to *in vivo* phenotype of the viruses and their mutants. At the molecular level, the situation is reversed. Namely, the processing kinetics of P123 of SINV (Fig. 5a) and RRV (Fig. 3a, b, and 4a) unambiguously demonstrated that the true counterpart of SINV_I538_ (termed “attenuated” in the Cruz et al. study [16]) is actually wt RRV-T48, while mutant RRV-T48_A532V_ represented a counterpart of SINV_T538_ (used as an example of wt SINV by Cruz et al.). In other words, two “attenuated” viruses (SINV_I538_ and RRV-T48_A532V_) that were compared in the earlier study actually possess opposite molecular phenotypes (fast and delayed processing of P123, respectively). Nevertheless, the major observation made by the authors of that previous study was also confirmed by us. This raises an intriguing question: why does the slowdown of P123 processing not increase type I IFN production in SINV? Our data suggest that for SINV infection in cell culture, the contribution of viral PAMPs dominates over the impact of replicase PAMPs. As in SINV, both Ile538 and Thr538 are naturally occurring P3 residues, it could be speculated that inhibition of STAT1/2 phosphorylation by SINV_T538_ that results in suppression of IFN signaling may represent a mechanism to counter the increase in the amount of replicase-synthesized PAMPs. The situation with RRV, particularly with RRV-T48_A534V_, is opposite: replicase PAMPs dominate over viral PAMPs. As the A534V (and A532V) substitutions are not naturally occurring in RRV, the virus apparently lacks specific mechanism(s) to counteract the elevated type I IFN production caused by these substitutions.

The link between P123 processing and replicase PAMP production suggests that neurovirulence of SINV and reduced virulence of RRV-T48_A534A_ may both be due to increased production replicase PAMP RNAs. It is likely that the restriction of virus infection (as observed for RRV) or the enhanced spread of infection leading to neurovirulence (as for SINV) depends on multiple additional factors such as sequence context (viral strain), production of viral PAMPs, presence or absence of mechanisms counteracting type I IFN signaling, and specific properties of infected tissue. It is also clear that milder suppression of P123 processing may result in a different effect compared to that caused by more prominent suppression (Fig. 4). If, as may be the case for SFV and SINV, slowdown of P123 processing indeed results in increased neurovirulence, it would have implications for the development of alphavirus protease inhibitors: if they were not potent enough to fully suppress virus replication, they could have unpredictable effects on virus infection.

Our results clearly indicate that the reduced *in vivo* pathology resulting from infection with RRV-T48_A534V_ is due to increased production of type I IFN-inducing RNAs (more specifically, replicase PAMP RNAs) and, consequently, also type I IFNs. The underlying molecular mechanism is altered processing of nonstructural polyproteins, but the overall outcome is modulated by various virus-host interactions, including production of different PAMP RNAs. Similar studies with New World alphaviruses will be useful to understand whether this type of IFN regulation (via nonstructural polyprotein processing) is conserved among different alphaviruses and contributes to New World virus neurovirulence.

## MATERIALS AND METHODS

Information on cells and viruses used is provided in File S1 in the supplemental material. Standard virology, molecular biology, immunology, and *in vivo* methods used in this study are also described in File S1 in the supplemental material.

10.1128/mBio.00044-18.1 TEXT S1  Supplemental methods. Download TEXT S1, DOCX file, 0.1 MB. Copyright © 2018 Liu et al. 2018 Liu et al. This content is distributed under the terms of the Creative Commons Attribution 4.0 International license.

### Analysis of virus-generated PAMP RNAs.

RNA samples obtained from infected cells were treated with DNase I (Roche, Germany) for 60 min in 37°C. For total RNA from RRV-T48- or RRV-T48_A534V_-infected cells (multiplicity of infection [MOI] of 10; 6 h postinfection [p.i.]), 100-µg aliquots were also incubated with biotinylated oligo(dT) probe, and the bound poly(A)^+^ fraction was removed using streptavidin MagneSphere paramagnetic particles (PolyATtract mRNA isolation systems kit; Promega, USA), resulting in poly(A)^−^ RNA samples. All samples were repurified with RNeasy kit (Qiagen, Germany) and eluted with 20 µl water. Prior to transfection of Cop5 cells, the infectious viral RNAs were UV inactivated for 5 min in a Hoefer UV crosslinker UVC5000. One microgram of total or poly(A)^−^ RNAs were used to transfect confluent cultures of Cop5 cells in 12-well plates using Lipofectamine 2000 reagent. Transfected cells were incubated at 37°C. At 24 h posttransfection (p.t.), the supernatants were collected and centrifuged at 3,000 × *g* for 5 min. The amount of IFN-β produced by the transfected cells was determined using VeriKine mouse IFN-β enzyme-linked immunosorbent assay (ELISA) kit (PBL Assay Science, USA).

### Analysis of type I IFN production by replicases of RRV and SINV.

Regions encoding replicase (nonstructural proteins [nsPs]) of RRV-T48, RRV-T48_A532V_, RRV-T48_A534V_, SINV_I538_, SINV_T538_ and SFV4 (used as positive control [10]) were cloned into the pMC-gtGTU2 vector (FIT Biotech Plc, Finland). The resulting plasmids (1 µg/transfection) were used to transfect confluent cultures of Cop5 cells in 12-well plates using Lipofectamine 2000 reagent. Transfected cells were incubated for 48 h at 37°C, after which IFN-β released into cell culture medium was measured as described above. For each independent experiment, the IFN-β levels in supernatants from cells transfected with RRV or SINV replicase expression plasmids were normalized to the amount of IFN-β produced by cells transfected by plasmid expressing SFV4 replicase.

### Statistical analysis.

Western blot band density, IFN bioassay, infiltrated-cell density, mouse weight, and plaque assay data were analyzed using two-way analysis of variance (ANOVA) with a Bonferroni *post hoc* test. IFN production data were analyzed using Student’s two-tailed unpaired *t* test. All data were tested and met normality using the D’Agostino-Pearson normality test prior to analysis except for differences in mouse clinical scores, which were analyzed using the nonparametric Mann-Whitney test. All statistical analyses were performed with GraphPad Prism software.
