# Supplementary figures and images for: Decreased Virulence of Ross River Virus Harboring a Mutation in the First Cleavage Site of Nonstructural Polyprotein Is Caused by a Novel Mechanism Leading to Increased Production of Interferon-Inducing RNAs
Source: mBio. 2018 Aug 21;9(4):e00044-18. doi: 10.1128/mBio.00044-18 (PMC6106088; doi:10.1128/mBio.00044-18)

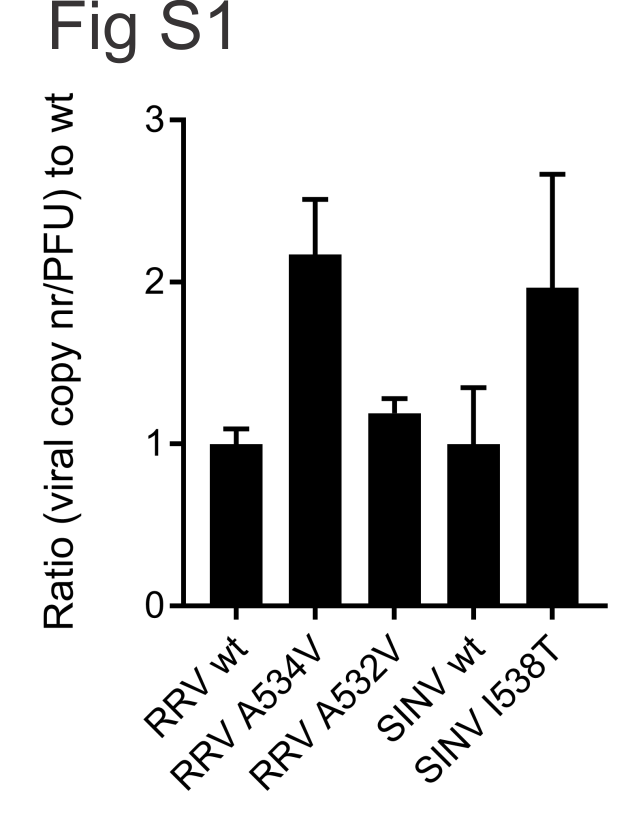

Supplement: FIG S1 [file mbo003183916sf1.tif]

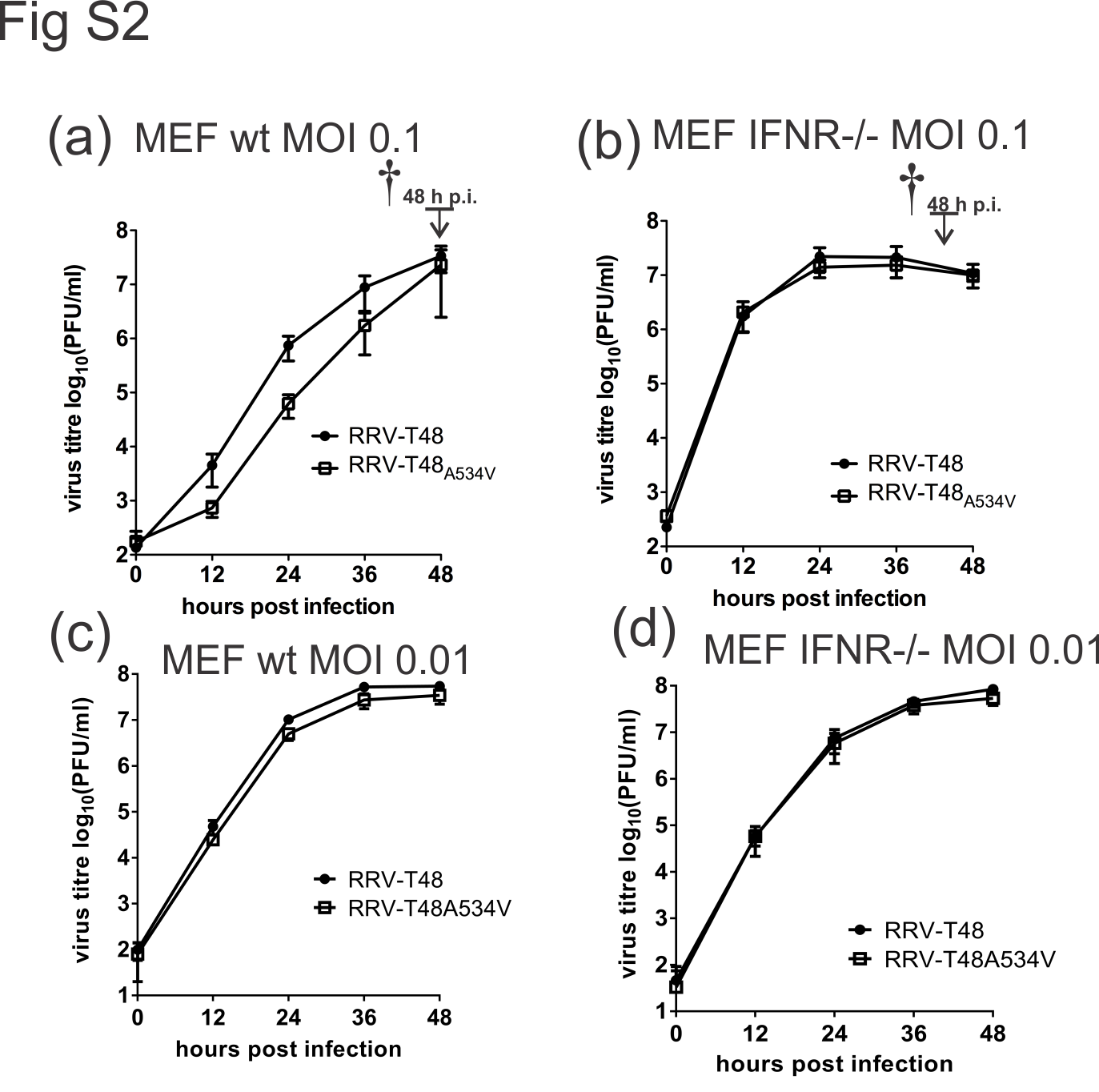

Supplement: FIG S2 [file mbo003183916sf2.tif]

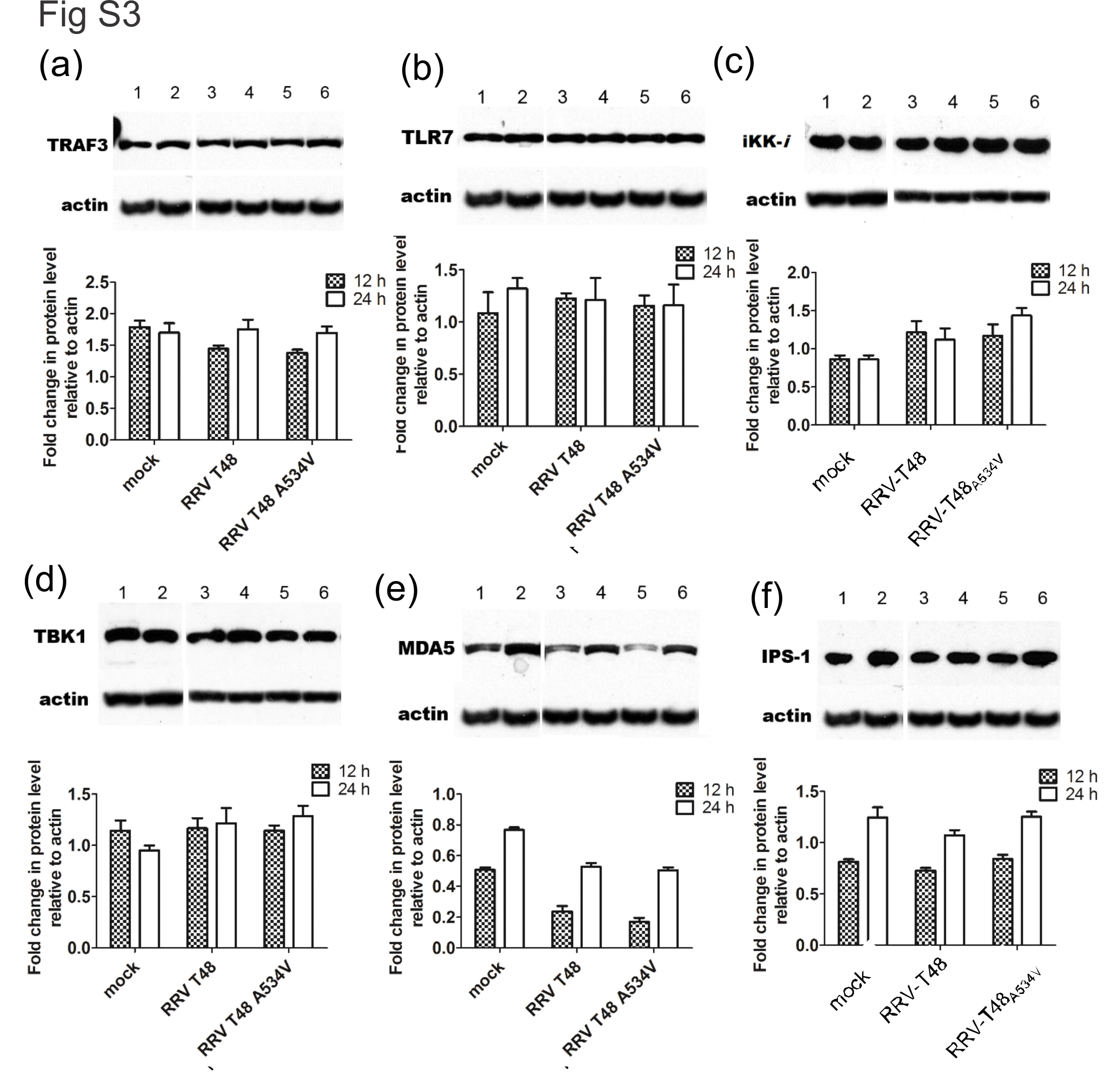

Supplement: FIG S3 [file mbo003183916sf3.tif]

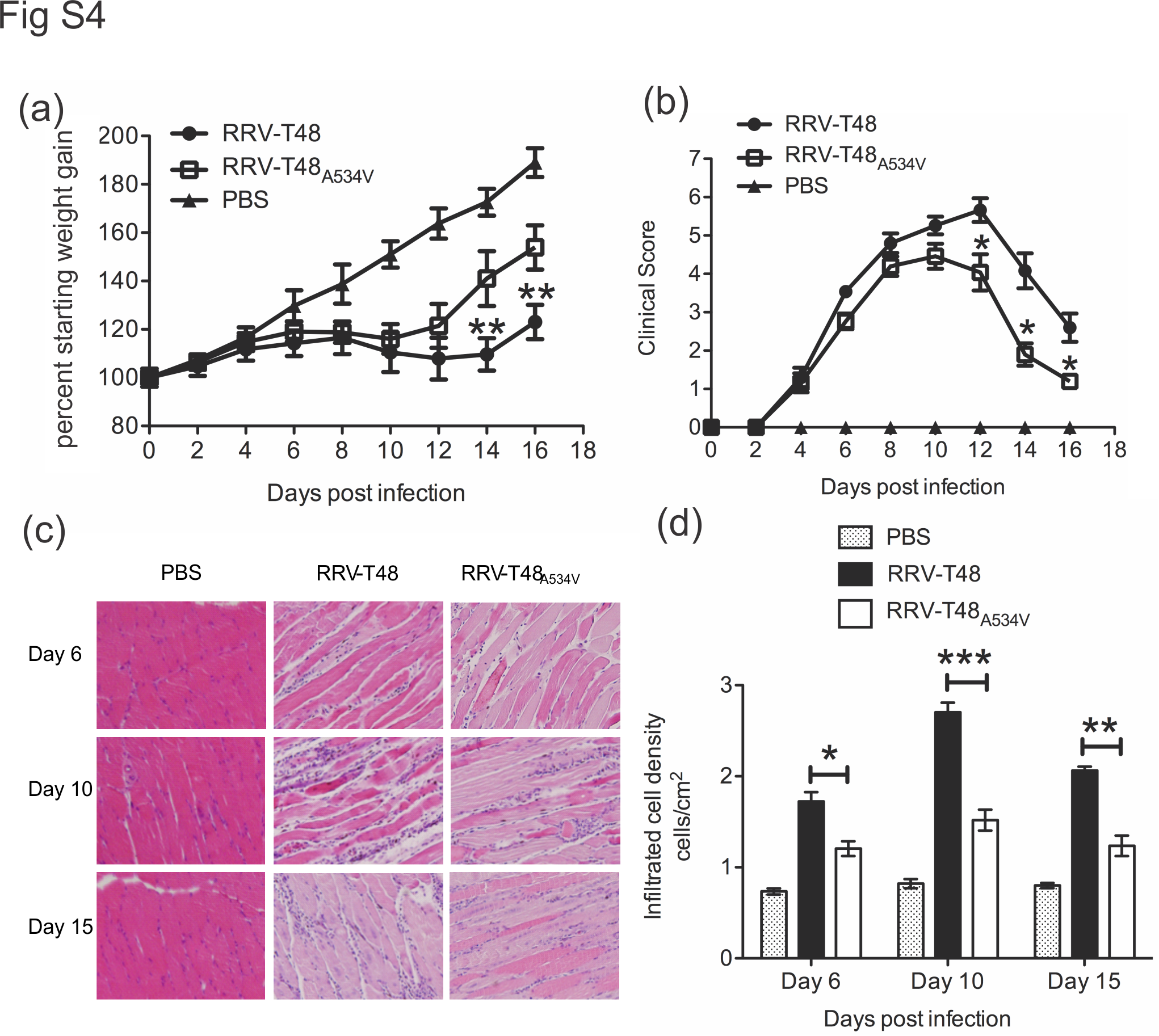

Supplement: FIG S4 [file mbo003183916sf4.tif]
