# Supplementary material for: Decreased Virulence of Ross River Virus Harboring a Mutation in the First Cleavage Site of Nonstructural Polyprotein Is Caused by a Novel Mechanism Leading to Increased Production of Interferon-Inducing RNAs
Source: mBio. 2018 Aug 21;9(4):e00044-18. doi: 10.1128/mBio.00044-18 (PMC6106088; doi:10.1128/mBio.00044-18)
Supplement: TEXT S1 [file mbo003183916s1.docx]

**Supplemental Methods**

**Cells and Viruses**

Vero and BHK-21 (C-13) cells (Sigma-Aldrich, USA) were propagated in Opti-MEM (Life Technologies, USA) supplemented with 5% fetal bovine serum (FBS, Bovogen). Cop5 (murine fibroblast) cells were maintained in Iscove's modified Dulbecco's medium (IMDM, Gibco, USA) containing 10% FBS and 2 mM L-glutamine. HeLa and mouse fibroblast L929 cells were cultivated using Dulbecco's modified Eagle's medium (DMEM, Gibco, USA) supplemented with 5% FBS. WT and INFAR^-/-^ MEFs were propagated in DMEM supplemented with 10% FBS. All cells were maintained at 37^o^C and 5% CO_2_ in the presence of 100 U/mL penicillin and 0.1 mg/mL streptomycin.

The infectious cDNA (icDNA) clone of prototype RRV-T48, ***a highly virulent Ross River virus strain***, was kindly provided by Richard Kuhn, Purdue University (1). The RRV-T48_A534V_ and RRV-T48_A532V_ mutants were generated by substituting a single nucleotide (positions 1679 and 1673, respectively) of RRV-T48 using PCR-based mutagenesis and subcloning procedures. This resulted in a change from Ala to Val at position 534 or at position 532 of nsP1. To reflect the naturally occurring nature of the introduced substitution, the laboratory-adapted strain TOTO1101 of Sindbis virus (SINV) (2), which has an Ile538 residue, is hereafter referred to as SINV_I538._ Virus designated as SINV_T538_ was generated by substituting a single nucleotide (position 1672) in the icDNA clone of TOTO1101; this resulted in a change from Ile to Thr at position 538 of nsP1. All virus stocks were generated by use of *in vitro* transcribed (mMACHINE^®^ SP6 Transcription Kit, Ambion, USA) infectious RNAs as previously described. Virus titers were determined by standard plaque assay on Vero cells (3). In addition, copy numbers of viral genomes in plaque-titered viral stocks were determined by extracting viral genomic RNA with Qiagen RNeasy Kit according to the manufacturer’s instructions. Viral genomic copy numbers in samples was determined with qRT-PCR using HOT FIREPol^®^ EvaGreen^®^ qPCR Supermix (Solis BioDyne, Estonia) according to manufacture instructions. Standard curves using serial dilutions of plasmids containing the target sequences were used to quantify the RNA in samples containing unknown number of viral genome copies. Sequences of the primers used in the assay were as follows: SINV 5’ GAC AGG CAC CGC AAG AAT GAC and 5’CGG CTG CGT AAA TGC CTG TAG; RRV 5’ CAA CGC GAA GGG CAC AGT C and 5’ GTC ACC GTG GAGA AAA TTC.

The presence of introduced mutations was confirmed by sequencing the 1/2 site region (nucleotides 1640 – 2500 of RRV-T48 genome). In addition, fragments obtained on the RRV-T48_A534V_ cDNA template were also cloned into pJet1.2 vector (Thermo Scientific, USA), and 10 randomly selected individual clones were sequenced.

**Infectious-centre assay**

For infectious-centre assays (ICAs), 8 × 10^6^ BHK-21 cells were electroporated with 3 μg in vitro-synthesized RNA. Ten-fold dilutions of transfected cells were seeded into 6-well tissue culture plates containing 1.5 × 10^6^ BHK-21 cells per well. After 4 h of incubation at 37°C, the cell culture medium was aspirated and the cells were overlaid with 2 ml DMEM supplemented with 2% FBS and 0.8% carboxymethyl cellulose (Sigma Life Science, USA). After 2 to 3 days of incubation at 37°C, plaques were stained with crystal violet. Efficiency of virus rescue was calculated as PFU per μg of viral RNA transcript.

**Multi- and single-step growth curves**

BHK-21, Vero, HeLa, L929, wt MEF and IFNAR^-/-^ MEF cells were grown on 24-well plates to 80% confluency and infected with RRV-T48 or RRV-T48_A534V_ at multiplicity of infection (MOI) 0.1 PFU/cell; for MEF cells, MOI 0.01 and 10 were also used. Aliquot of supernatant was harvested immediately after addition of growth media (time point “0”); supernatants were collected at 12, 24, 36 and 48 h post infection (h p.i.) for cells infected at an MOI 0.1 or 0.01 and at 3, 6, 9, 12 and 24 h p.i. for cells infected at an MOI 10. Each time point was an independent infection; the amount of infectious virus in the samples was determined using plaque assay as previously described (3).

**IFN-β treatment and transfection of cells with poly(I:C)**

Vero cells seeded into 12-well plates were infected with RRV-T48 or RRV-T48_A534V_ at an MOI 0.1. After 1 h adsorption, serum-free medium containing 0, 100, 250 or 500 pg/ml human IFN-β (PeproTech, USA) was added to the cells. Plates were incubated at 37^o^C for 24 h. Growth inhibition was calculated by dividing virus titer from untreated sample with virus titer from IFN-treated samples. Transfection of L929 cells with 10µg polyinosinic:polycytidylic acid (poly(I:C), Sigma-Aldrich) was carried out using Lipofectamine^®^ 2000 (Life Technologies) reagent according to manufacturer’s protocol; cells were incubated at 37^o^C for 6 h. Culture media were replaced with complete growth media and the cells were incubated at 37^o^C for a further 6 or 18 h.

**Detection of type I IFNs by bioassay**

The concentration of type I IFN in cell culture supernatants was assessed by a cytopathic effect (CPE) inhibition bioassay using Semliki Forest virus (SFV) infection of L929 cells (4). Briefly, UV inactivated and serially diluted samples were added to the L929 cells and cultured for 24 h. SFV (2.5 × 10^3^ PFU) was added to each well and 48 h later CPE was determined by crystal violet staining. The crystal violet was released by adding 100 μl of 100% methanol to each well, and the absorbance was measured at a wavelength of 595 nm. The type I IFN concentrations in international units (IU) per ml were calculated according to the protection against SFV infection provided by treatment with known concentrations of human IFN-β (Sigma-Aldrich). The sensitivity of the assay was 10 IU per ml.

**SDS-PAGE and Western blot analysis**

Cells were lysed in RIPA buffer (150 mM NaCl; 0.1% Triton X-100; 0.5% sodium deoxycholate; 0.1% SDS; 50 mM Tris-HCl pH 8.0; 1% protease inhibitor cocktail, Sigma-Aldrich) and proteins were separated by SDS-PAGE followed by transfer onto a PVDF membrane (Millipore, USA). Membranes were probed using the following antibodies (Santa Cruz Biotechnology Inc): RIG-I (H-300, cat#sc-98911); MDA5 (H-61, cat#sc-134513); IPS-1 (E-3, sc-166583); TRAF3 (H-122, cat#sc-1828); IRF7 (H-246, cat#sc-9083); TLR7 (4F4, cat# sc-57463); MyD88 (B-1, cat# sc-136970); TBK1 (108A429, cat# sc-52957); ikk-*i* (A11, cat#sc-376114). Proteins were visualized using enhanced chemiluminescence (Amersham^TM^ ECLTM, USA) according to the manufacturer’s instructions. Protein band density analyses were performed using Image J software.

***In vitro* transcription/translation and immunoprecipitation**

*In vitro* transcription and translation were carried out using the TNT-coupled SP6 rabbit reticulocyte lysate system (Promega, USA) according to the manufacturer's protocol. Reaction mixtures (25 μl) containing 10 μCi of [^35^S]Met (Perkin-Elmer, USA) and 1 μg of a plasmid containing icDNA of RRV-T48, RRV-T48_A534V_, RRV-T48_A532V_, SINV_I538_ or SINV_T538_ were incubated for 1 h at 30°C; translation was stopped by adding cycloheximide to a final concentration of 1 mM. RNase A was added at a final concentration of 10 ng/μl, and the mixtures were incubated for another 5 min. Samples were denatured by heating in SDS gel-loading buffer, separated by SDS-PAGE, and visualized using a Typhoon imager (GE Healthcare, USA).

For immunoprecipitation, products of *in vitro* transcription/translation and processing reactions for RRV-T48 and RRV-T48_A534V_ were denatured by boiling in LDS-NET150 buffer (50 mM Tris-HCl pH 8.0, 2% lithium dodecyl sulphate, 150 mM NaCl, 5 mM EDTA, and 1% NP-40), diluted 100-fold with NET150 buffer (50 mM Tris-HCl pH 8.0, 150 mM NaCl, 5 mM EDTA, and 1% NP-40), and incubated overnight at 4°C with rabbit polyclonal antiserum against SFV nsP1, SFV nsP2 or SFV nsP3 generated in house. Immunocomplexes were precipitated with protein A-Sepharose CL-4B (Sigma-Aldrich) for 1 h at 4°C and then washed four times with NET buffer containing 400 mM NaCl. The precipitated proteins were denatured by heating in SDS gel-loading buffer, separated by SDS-PAGE, and visualized using a Typhoon imager.

**Analysis of translational shutoff in RRV-T48 and RRV-T48_A534V_ infected cells**

Metabolic labeling of proteins in virus-infected cells was performed as previously described (5). Briefly, confluent BHK-21 cells grown on 12-well plates were infected at an MOI 10 with RRV-T48 or RRV-T48_A534V_. After an incubation period of 1 h, the infected cells were overlaid with complete medium. At selected time points (4, 6, 8, 10, 12, 16, 20 and 24 h p.i.), cells were washed with PBS, starved for 30 min in Met- and Cys-free DMEM, and labeled for 30 min with media containing 50 µCi [^35^S]Meth and [^35^S]Cys (Perkin Elmer). After labeling, cells were washed with PBS, lysed in 50 µl SDS gel-loading buffer and boiled for 5 min. Lysates corresponding to 50,000 cells were subjected to 10% SDS-PAGE. Gels were subsequently dried and submitted to autoradiography.

**Total RNA extraction**

8×10^6^ BHK-21 cells were infected with RRV-T48, RRV-T48_A532V_, RRV-T48_A534V_, SINV_I538_ or SINV_T538_ at an MOI 10. Alternatively, cells were infected with RRV-T48 or RRV-T48_A534V_ at an MOI 1. Cells were collected at 4 h or 6 h p.i. (SINV_I538_, SINV_T538_), 6 h or 8 h p.i. (RRV-T48, RRV-T48_A532V_, RRV-T48_A534V_ at an MOI 10) or 6 h p.i. (RRV-T48, RRV-T48_A534V_ at an MOI 1). Total RNA was extracted using TRIzol Reagent (Life Technologies, USA) according to the manufacturer’s protocol; RNA isolated from mock-infected cells was used as control.

**Northern blotting**

For detection of viral negative strand RNA, 10 µg of total RNA isolated from infected

BHK-21 cells was used, while for detection of positive strand RNAs the amounts of total RNA used were 5 µg for RRV or 2 µg for SINV. Samples were prepared as described previously (6). Digoxigenin (DIG)-labelled RNA probes complementary to residues 11480-11820 of RRV genome or residues 10771-11087 of SINV genome were used for detection of positive strand RNAs; probes corresponding to residues 8934-9379 of RRV genome or residues 2289-2750 of SINV genome were used for detection of negative strands. All probes were generated using a DIG hybridization kit (Roche, Germany). Filters were hybridized overnight, and blots were washed and developed according to the manufacturer's protocols (Roche, Germany).

**Mouse infections studies**

***Immunocompetent*** ***inbred*** C57BL/6 mice were obtained from the Animal Resources Centre (Perth, Australia) and bred in-house. IFNAR^-/-^ mice on a C57BL/6 background were obtained from the University of Queensland (Brisbane, Australia) and bred in-house. Groups of twenty-day-old C57BL/6 wt or IFNAR^-/-^ mice (5 animals per group) were inoculated subcutaneously (s.c.) in the thorax below the right forelimb with 10^4^ PFU of RRV-T48 or RRV-T48_A534V_ diluted in PBS to a volume of 50 μl (7). Mock-infected mice were inoculated with PBS only. Wt mice were weighed and scored for disease daily. RRV disease scores were assessed as described previously (7). In survival experiments, animals were monitored every 6 h during the first 24 h p.i. and every 2 h thereafter. Humane end point was defined as a clinical score of 7 or weight loss greater than 15% of starting weight (7). Animals that reached experimental end points were euthanized. For one group of wt and one group of IFNAR^-/-^ mice, serum, spleens and quadriceps were collected at 24 h p.i.; viral titers in these samples were assessed by plaque assay. Animal experiments were approved by the Animal Ethics Committee of Griffith University (Gly/01/14/AEC).

**Histological analysis**

Mouse quadriceps were collected and fixed in 4% paraformaldehyde (PFA), followed by paraffin embedding. Samples were cut into five-micron sections and stained with haematoxylin and eosin (H&E). Images were taken using a Nikon TI-E microscope, and density of the infiltrated cells was analyzed using Image J software.

**References**

1. Kuhn RJ, Niesters HG, Hong Z, Strauss JH. 1991. Infectious RNA transcripts from Ross River virus cDNA clones and the construction and characterization of defined chimeras with Sindbis virus. Virology 182:430–441.

2. Polo JM, Davis NL, Rice CM, Huang HV, Johnston RE. 1988. Molecular analysis of Sindbis virus pathogenesis in neonatal mice by using virus recombinants constructed in vitro. J Virol 62:2124–2133.

3. Lidbury BA, Simeonovic C, Maxwell GE, Marshall ID, Hapel AJ. 2000. Macrophage-Induced Muscle Pathology Results in Morbidity and Mortality for Ross River Virus-Infected Mice. J Infect Dis 181:27–34.

4. Liu WJ, Wang XJ, Clark DC, Lobigs M, Hall RA, Khromykh AA. 2006. A Single Amino Acid Substitution in the West Nile Virus Nonstructural Protein NS2A Disables Its Ability To Inhibit Alpha/Beta Interferon Induction and Attenuates Virus Virulence in Mice. J Virol 80:2396–2404.

5. Karo-Astover L, Šarova O, Merits A, Žusinaite E. 2010. The infection of mammalian and insect cells with SFV bearing nsP1 palmitoylation mutations. Virus Res 153:277–287.

6. Utt A, Das PK, Varjak M, Lulla V, Lulla A, Merits A. 2015. Mutations Conferring a Noncytotoxic Phenotype on Chikungunya Virus Replicons Compromise Enzymatic Properties of Nonstructural Protein 2. J Virol 89:3145–3162.

7. Herrero LJ, Nelson M, Srikiatkhachorn A, Gu R, Anantapreecha S, Fingerle-Rowson G, Bucala R, Morand E, Santos LL, Mahalingam S. 2011. Critical role for macrophage migration inhibitory factor (MIF) in Ross River virus-induced arthritis and myositis. Proc Natl Acad Sci 108:12048–12053.
